# Novel Application of Stem Cell-Derived Neurons to Evaluate the Time- and Dose-Dependent Progression of Excitotoxic Injury

**DOI:** 10.1371/journal.pone.0064423

**Published:** 2013-05-14

**Authors:** Ian M. Gut, Phillip H. Beske, Kyle S. Hubbard, Megan E. Lyman, Tracey A. Hamilton, Patrick M. McNutt

**Affiliations:** United States Army Medical Research Institute of Chemical Defense, Aberdeen Proving Ground, Maryland, United States of America; Univ. Kentucky, United States of America

## Abstract

Glutamate receptor (GluR)-mediated neurotoxicity is implicated in a variety of disorders ranging from ischemia to neural degeneration. Under conditions of elevated glutamate, the excessive activation of GluRs causes internalization of pathologic levels of Ca^2+^, culminating in bioenergetic failure, organelle degradation, and cell death. Efforts to characterize cellular and molecular aspects of excitotoxicity and conduct therapeutic screening for pharmacologic inhibitors of excitogenic progression have been hindered by limitations associated with primary neuron culture. To address this, we evaluated glutamate-induced neurotoxicity in highly enriched glutamatergic neurons (ESNs) derived from murine embryonic stem cells. As of 18 days in vitro (DIV 18), ESNs were synaptically coupled, exhibited spontaneous network activity with neurotypic mEPSCs and expressed NMDARs and AMPARs with physiological current:voltage behaviors. Addition of 0.78–200 μM glutamate evoked reproducible time- and dose-dependent metabolic failure in 6 h, with a calculated EC_50_ value of 0.44 μM at 24 h. Using a combination of cell viability assays and electrophysiology, we determined that glutamate-induced toxicity was specifically mediated by NMDARs and could be inhibited by addition of NMDAR antagonists, increased extracellular Mg^2+^ or substitution of Ba^2+^ for Ca^2+^. Glutamate treatment evoked neurite fragmentation and focal swelling by both immunocytochemistry and scanning electron microscopy. Presentation of morphological markers of cell death was dose-dependent, with 0.78–200 μM glutamate resulting in apoptosis and 3000 μM glutamate generating a mixture of necrosis and apoptosis. Addition of neuroprotective small molecules reduced glutamate-induced neurotoxicity in a dose-dependent fashion. These data indicate that ESNs replicate many of the excitogenic mechanisms observed in primary neuron culture, offering a moderate-throughput model of excitotoxicity that combines the verisimilitude of primary neurons with the flexibility and scalability of cultured cells. ESNs therefore offer a physiologically relevant platform that exhibits characteristic NMDAR responses, and appears suitable to evaluate molecular mechanisms of glutamate-induced excitotoxicity and screen for candidate therapeutics.

## Introduction

Excessive stimulation of central nervous system (CNS) neurons by excitatory neurotransmitters results in Ca^2+^ overload and cell death [1]. Glutamoreceptive neurons are highly abundant within the CNS, and over-activation of glutamate receptors (GluRs) is a common modality of excitogenic injury that is implicated in a variety of CNS disorders and neural degenerative disease [2]–[5]. The ionotropic GluRs (iGluRs) are classified into three groups based on their pharmacology and structural properties: NMDA receptors (NMDARs); AMPA receptors (AMPARs); and kainate receptors (KARs). AMPAR and KAR primarily allow Na^+^ influx, whereas NMDAR is a coincidence-gated, high-conductance Ca^2+^ channel that is both ligand-gated and voltage-dependent. Ionotropic GluR activity is dynamically regulated by several factors, including expression level and subunit composition [6]. While all three receptor groups have functional roles in neurotransmission and synaptic plasticity, synaptic NMDAR activity is primarily associated with plasticity, whereas AMPAR and, to a lesser extent KAR, mediate post-synaptic depolarization and neurotransmission [7]. Under conditions of elevated extracellular glutamate, the influx of Ca^2+^ through NMDARs is believed to elicit pathogenesis through activation of Ca^2+^-dependent proteases, altered phosphoproteomes, mitochondrial dysfunction, bioenergetic failure and cytosolic release of pro-apoptotic enzymes. At sufficiently high doses of glutamate, this process culminates in excitogenic cell death [1].

Treatments to mitigate neuronal damage during excitotoxic injury remain elusive, in part because of an incomplete understanding of the cellular processes initiated by excessive iGluR activation. Attempts to elucidate the mechanistic underpinnings of excitotoxicity in primary neurons have resulted in inconsistent findings, suggesting that variability in the origin, handling or treatment of primary cultures may influence experimental outcomes [8]–[10]. In addition to sample variability, primary neuron use is limited by ethical, technical and regulatory constraints, restricting the ability of many labs to elucidate how differences in culture or handling may affect excitogenic progression.

We have previously shown that highly enriched cultures of glutamatergic neurons (ESNs) derived from suspension-cultured murine embryonic stem cells exhibit developmental, functional, transcriptional and morphological characteristics of primary neurons, and are responsive to a variety of neurotropic stimuli [11], [12]. In contrast to most primary neuron cultures and neuroblastoma cell lines, differentiation of ESN reproducibly generates a glutamatergic neuron subtype with minimal contamination by GABAergic neurons and glial cells [12]. Hypothesizing that ESNs may offer a new model for excitotoxic injury, we first evaluated the functional expression of post-synaptic ionotropic glutamate receptors using electrophysiology and pharmacological agonists/antagonists. We then characterized the consequences of glutamate treatment on neuron morphology, gene expression and time- and dose-dependent appearance of neurotoxic markers. To evaluate the potential for therapeutic screening, we assessed the effect of several small molecule antagonists on excitotoxicity in a moderate-throughput format. The findings suggest that stem cell-derived neurons comprise a sensitive platform for excitotoxicity research that faithfully replicates neurotypic responses to excitogenic stimuli while offering the scalability, genetic tractability and flexibility of cultured cell lines.

## Materials and Methods

### Reagents

Mono-sodium glutamate, 2-amino-3-(5-methyl-3-oxo-1,2- oxazol-4-yl)propanoic acid (AMPA) kainic acid (KA), γ-aminobutyric acid (GABA), N-methyl-D-aspartate (NMDA), gadolinium chloride, and saponin were purchased from Sigma-Aldrich (St. Louis, MO). Solutions were diluted to the indicated concentrations in the described buffer at the time of the experiment. Fluo-4, Hoechst 33342, propidium iodide (PI) and PrestoBlue were purchased from Life Technologies (Carlsbad, CA) and prepared per the manufacturer's instructions. During time-lapse imaging, neurons were maintained in basal electrophysiologic buffer (BEB; in mM, 10 glucose, 1 MgCl_2_, 10 HEPES, 2 CaCl_2_, 3 KCl, 136 NaCl and 0.1% BSA, pH 7.4, 310±10 mOsm; Sigma-Aldrich).

### Embryonic stem cell culture and neuronal differentiation

Murine embryonic stem cells were maintained and differentiated into ESNs as previously described [12], [13]. Briefly, 3.5×10^6^ suspension-adapted ES cells were transferred to 25 mL of differentiation medium (Knockout DMEM with 10% ES qualified fetal calf serum, non-essential amino acids and 10 mM β-mercaptoethanol [Life Technologies]) in a 10-cm low-attachment culture dish (Corning, Lowell, MA) and maintained on a rotary shaker at 30 rpm at 37°C, 5% CO2 and 90% relative humidity. Complete media changes were conducted at 48 h intervals, and differentiation medium was supplemented with 6 μM retinoic acid (Sigma-Aldrich) after 4 and 6 days. On day 8 (DIV 0), cell aggregates were dissociated and plated on poly-D-lysine coated surfaces in Neurobasal-A with N2 vitamins (Invitrogen). At DIV 2 and beyond, ESNs were maintained in Neurobasal-A with B27 vitamins (Invitrogen). Experiments were conducted on ESNs at DIV 18–24. During the course of this study, data was collected from 13 independent ESN differentiations over 6 months, with no apparent change in results.

### Electrophysiology

DIV 18–24 neuron cultures were visualized on an Olympus IX51 microscope (Shinjuku, Tokyo, Japan) equipped with a 40x lens with differential interference contrast optics. 5–7 MΩ pipettes were pulled from capillary glass (Sutter Instruments, Novato, CA), backfilled with intracellular recording buffer and dipped in Sigmacote® (Sigma) just prior to use. Electrophysiology data was acquired at 20–22°C with an EPC10 (Heka, Lambrecht/Pfalz, Germany) and Heka Patchmaster 2.53 software. Data analysis and graphing was performed in Heka Fitmaster 2.53, Igor Pro v6 (Wavemetrics, Portland, OR) and Prism v6 (Graphpad Software, La Jolla, CA). The 10–90% rise time, peak amplitude, and decay kinetics of mEPSCs were calculated with MiniAnalysis (Synaptosoft, Inc, Decatur, GA).

For characterization of intrinsic voltage-gated responses, sEPSCs and current clamp recordings, pipettes were filled with an intracellular recording buffer containing (in mM): 140 K-gluconate, 5 NaCl, 2 Mg-ATP, 0.5 Li-GTP, 0.1 CaCl_2_, 1 MgCl_2_, 1 ethylene glycol-bis (b-aminoethyl ether) –*N,N,N,N*-tetraacetic acid (EGTA) and 10 HEPES. Cultures were bathed in an extracellular recording buffer (ERB) containing (in mM): 140 NaCl, 3.5 KCl, 1.25 NaH_2_PO_4_, 2 CaCl_2_, 1 MgCl_2_, 10 Glucose, and 10 HEPES. All buffers were adjusted to pH of 7.3 with NaOH and an osmolarity of 315±10 mOsm with glucose prior to recording.

To characterize current:voltage (I-V) relationships and mEPSC kinetics, neurons were bathed in ERB supplemented with 5 µM TTX and 1 µM glycine and patched with electrodes containing (in mM): 125 CsCH_4_SO_3_, 4 NaCl, 1 MgCl_2_, 3 KCl, 9 EGTA and 8 HEPES (pH 7.3, adjusted with CsOH). TTX and cesium were used to block Na^+^ and K^+^ channel currents, respectively. For characterization of mEPSCs, neurons were patched and recorded in voltage-clamp mode at −80×mV. For I-V curves, neurons were held at −70 mV and stepped from −100 mV to +60 mV in regular increments. At each potential, neurons were sequentially perfused with (i) ERB for 15 sec; (ii) ERB with 50 µM NMDA or 100 uM kainate for 10 sec; and (iii) ERB for 35 sec to restore a baseline response. Perfusions were conducted using a three barrel Fast Step system (Warner Instruments, Hamden, CT). I-V responses were determined by subtracting the current measured during the initial ERB perfusion from peak currents during administration of agonist. Currents were re-measured during the washout step and compared to the baseline readings to confirm that the response was fully reversible and verify the integrity of the patch. For measurement of Mg^2+^-free ERB NMDAR currents, the perfusate was formulated as above, but without Mg^2+^. For AMPAR I-V curves, Kainate (100 uM; KA) was used as a non-desensitizing agonist of AMPAR channels to avoid neurotoxicity induced by simultaneous application of AMPA and cyclothioheximide [14], [15].

### Immunoblotting

ESN cultures were lysed with denaturing cell extraction buffer (Life Technologies) and harvested by scraping. Lysates were vortexed briefly, stored at 4°C for 15 min, and clarified by centrifugation for 3.5 min at 16,000×g. Total protein concentration was determined by bicinchoninic acid analysis (Thermo Scientific, Rockford, IL), and 25 µg of total protein were separated on a 4–12% Nupage gel (Life Technologies). Gels were transferred to PVDF and probed with primary antibodies against GluN1, GluN2B, GluN1A/B, GluA1–4 and GluR6 (Synaptic Systems, Goettingen, Germany) diluted 1∶1000 in TBS with 0.05% Tween-20 (TBST; Life Technologies). Proteins were visualized with goat anti-mouse or goat anti-rabbit Alexa-488 labeled antibodies diluted 1∶2500 in TBST and imaged with a Versadoc MP4000 (Biorad, Hercules, CA).

### Time-lapse microscopy

Images were collected on a Zeiss LSM-700 confocal microscope with a constant-temperature environmental chamber. For Fluo-4 staining, ESNs on 18-mm coverslips were stained and mounted as previously described [13], and maintained at 37°C until imaged at 63x using manufacturer-specified laser excitation wavelength and emission filter sets. Although soma and axons exhibited commensurate changes in Ca^2+^ response, axons proved to have a higher signal:noise ratio and were subsequently used. Due to the high degree of fasciculation (see [11]), it was not possible to distinguish of axons from dendrites in the proximity of the cell body, so imaging was conducted in distal processes to restrict the analysis to axons. Zen 2009 (Carl Zeiss, Inc) was used to determine the mean fluorescence intensity over a full resolution field of view for up to 600 sec. The data were normalized to the change in fluorescence relative to baseline values (ΔF/F_0_) via the following equation: *y =  (F_peak_ − F_0_)/F_0_*. To ensure representative comparisons between controls and experimental conditions, all values collected were within the linear dynamic range of detection.

### Immunofluorescence and scanning electron microscopy

ESNs on 18-mm coverslips were incubated in NBA supplemented with vehicle or described concentrations of glutamate for indicated durations. For immunocytochemistry, neurons treated with vehicle or 12.5 μM glutamate were fixed in 4.0% paraformaldehyde in PBS (Life Technologies) at 2, 6, or 24 h, permeabilized in 0.1% saponin (Sigma), blocked with 3% BSA (Sigma), and probed with anti-NR2A/B, anti-TAU antibody, anti-MAP2 antibody (Synaptic Systems) or anti-GluR1 antibody (Epitomics, Burlingame, CA). Proteins were visualized with Alexa Flour-labeled goat anti-mouse, anti-rabbit, and anti-guinea pig antibodies. Coverslips were mounted onto glass slides with Prolong Gold anti-fade reagent containing DAPI (Life Technologies). Images were collected on a Zeiss LSM-700 confocal microscope with a 40x objective. Intensities were enhanced after image capture to visualize neurite integrity. Quantification of neuronal fragmentation and varicosity formation was done with NIH ImageJ. For scanning electron microscopy (SEM), neurons exposed to 200 μM glutamate or vehicle for 6 h were fixed for 30 min with buffered 1.6% paraformaldehyde/2.5% glutaraldehyde. Following fixation, samples were osmicated and processed by standard dehydration protocol to critical point drying. Dried samples were ion beam-coated with gold/palladium and imaged using a JEOL JSM-7401F field emission scanning electron microscope.

### Endpoint evaluation of DNA condensation and membrane disruption

ESNs plated on 18-mm coverslips were washed and stained with 5 µg/mL Hoechst and 5 µg/mL PI in NBA for 10 and 5 min, respectively, at 37°C, washed, fixed in 4.0% paraformaldehyde PBS pH 7.4, and mounted onto glass slides with Prolong Gold antifade reagent. Coverslips were imaged on a Zeiss LSM-700 confocal microscope at 40x and 63x using manufacturer-specified laser excitation wavelengths and emission filter sets. Necrosis and apoptosis were evaluated per the following. Healthy neurons had large nuclei with a diffuse chromatin morphology colored blue by Hoechst 33342. Apoptotic nuclei exhibited condensed Hoechst-stained chromatin, while necrosis resulted in PI uptake. Glial cells (∼5–10% per cover slip) were identified by light microscopy and excluded from analyses. For quantitation of nuclear size, orthogonal measurements of nuclear diameter were made using Zen 2007, averaged, and used to calculate the two-dimensional nuclear area.

### Viability measurements

ESNs were plated in 24-well, 48-well or 96-well dishes and treated per indicated conditions. To quantify metabolic activity, PrestoBlue (Invitrogen) was added to ESNs at a final 1x concentration in fresh NBA-B27 and ESNs were incubated for an additional 45 min at 37°C. Metabolic conversion of PrestoBlue to a fluorescent product was measured with a Synergy MX plate reader (Biotek, Winooski, VT) using excitation of 535 nm and emission of 595 nm. To evaluate potential glial contributions to metabolic activity, wells were treated with 5 mM glutamate subsequent to measurements of metabolic activity, and fluorescence intensities were subtracted from experimental wells. Microscopic evaluation of cultures confirmed that addition of 5 mM glutamate did not induce any detectable toxicity in the rare glia, whereas all neurons exhibited PI uptake or nuclear condensation within 2 h. Using this approach we found that glia contributed less than 5% of the metabolic activity measured in untreated controls. Since glial contamination did not appear to significantly affect measurements of toxicity, it was not used for subsequent viability assays.

For pulsed exposures, supplements prepared in BEB or in modified BEB as described were added at the described concentration for 5 min, washed three times with NBA-B27 and returned to the incubator for 6 or 24 h before determining viability. EC_50_ values were calculated by fitting the dose-response curve to a four-parameter sigmoidal model using Prism v5.04 (Graphpad Software, La Jolla, CA). For prophylactic screening, a cocktail of neurotrophic factors (NTFs; 10 ng/mL human neurotrophin-3 [NT3], 10 ng/mL human brain-derived neurotrophic factor [BDNF], 10 ng/mL rat glial-derived neurotrophic factor [GDNF] and 25 ng/mL rat ciliary neurotrophic factor [CNTF]; R&D Systems, Minneapolis, MN) was added 18 h prior to and concurrent with the addition of glutamate.

### RNA analyses

RNA was extracted from ESNs using the RNeasy Mini Kit (Qiagen, Germantown, MD) and RNA quality was assessed using the NanoDrop 2000c UV-Vis spectrophotometer (Thermo Scientific). Expression profiling using next-generation sequencing (RNAseq) was conducted as previously described [11], [16]. Reads were assembled, annotated and screened for differential expression of *Gria2* isoforms using the Seqman Pro software package (DNAStar, Inc, Madison, WI). Library sizes were normalized using the DESeq *normalizedCounts* function and normalized reads are presented as pseudocounts.

For quantitative, real-time reverse-transcription PCR (QPCR), cDNA was synthesized using the RT^2^ First Strand Kit (SaBiosciences, Frederick, MD) according to manufacturer's protocol). QPCR was performed using a BioRad-CFX 96-well Real-Time System with iQ SYBRGreen Supermix in a PTC-100 MJ Research thermal cycler (Waltham, MA). Primer sequences were from the Harvard University Medical School Primer Bank (Table S1) [17]. PCR conditions were 95°C for 3 min, followed by 39 cycles of 95°C for 10 sec, 54°C for 10 sec, and 72°C for 30 sec, then a melt curve from 54 to 95°C in 0.5°C increments. The ΔC_T_ for control and treated samples were normalized to β3-tubulin to determine the normalized log_2_ fold change.

### Statistics

Statistical significance among means was determined utilizing repeated-measures ANOVA and *P* values were calculated with the Tukey's post-hoc test. For binary comparisons of means, the Student's t-test was used. Unless otherwise stated, all quantitative data are presented as mean plus/minus one standard deviation, with the following markers of statistical significance: * indicates a *P*<0.05; ** indicates a *P*<0.01; *** indicates a *P*<0.001.

## Results

### Electrophysiologic characterization of DIV 18–24 ESNs

**Figure 1:**
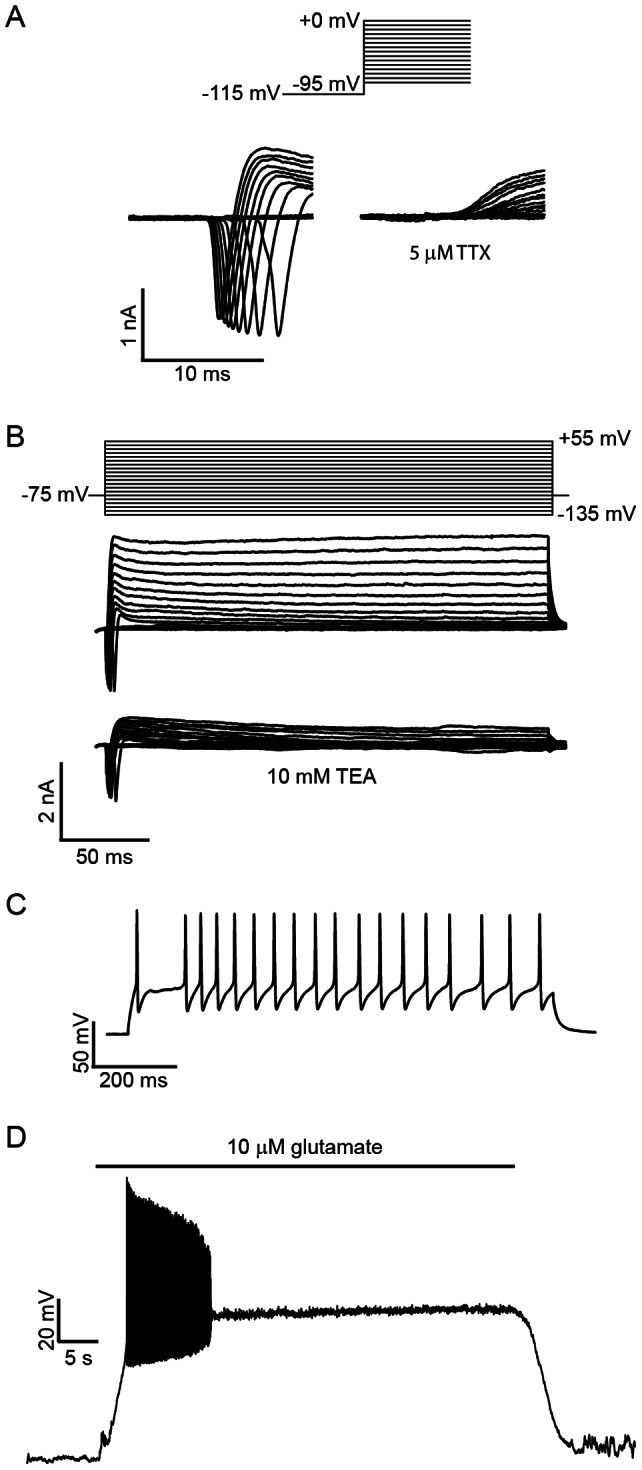
Representative whole cell patch clamp recordings demonstrating elicited electrical responses in DIV 18+ ESNs. A. Voltage-step protocol demonstrating a fast-activating, fast-inactivating inward Na^+^ current that is inhibited by addition of TTX. Vm  = −75 mV. B. Voltage-step protocol demonstrating a delayed rectifier K^+^ current that is inhibited by addition of TEA. Vm  = −75 mV. C. Current clamp recordings showing repeated overshooting action potentials are evoked by injection of a 75 pA current. D. Voltage-clamp recordings showing action potential burst and sustained depolarization block following addition of 10 µM glutamate. Voltages are adjusted for a liquid junction potential of −15 mV.

To explore the possibility that stem cell-derived glutamatergic neurons might serve as a model of excitotoxicity, we adapted a method of neuronal differentiation from feeder-cell free suspension monocultures of ESCs [11]. Starting at eighteen days after plating (DIV 18; corresponding to 26 days after the start of differentiation), developmental stage IV/V ESNs were evaluated for neuronal activity using the whole-cell recording configuration. After adjustment for liquid junction potential, the resting membrane potential (RMP) was measured at −84.5±4.5 mV (mean ± standard deviation; n = 64). Voltage-clamp recordings revealed a fast-activating, fast-inactivating, TTX-sensitive inward sodium current between −45 and −55 mV (mean  = 48.9±4.2) (Figure 1A) and an outward TEA-sensitive current typical of a delayed rectifier potassium conductance (Figure 1B). Repeated overshooting action potentials were produced in response to depolarizing current pulses (Figure 1C). Bath application of 10 µM glutamate evoked a series of action potentials lasting approximately 5–10 seconds, followed by a depolarization block that persisted until glutamate washout (n = 14; Figure 1D). Finally, inhibitory post-synaptic currents were rarely observed, consistent with transcriptomic and proteomic data that ESNs are predominantly glutamatergic [11], [12].

### Transcriptional and proteomic characterization of iGluR expression

**Figure 2:**
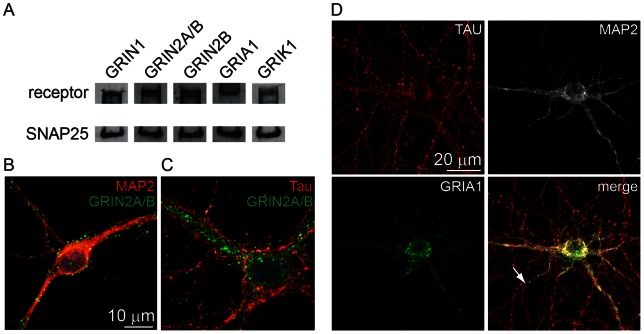
Expression of GluRs. A. DIV 16 ESN lysates were evaluated for the presence of select GluRs by immunoblot. SNAP-25 is present as a measure of GluR relative abundance to a highly expressed neuronal protein. B,C. ESNs were evaluated for the compartmentalization of GRIN2A/B (green; B, C) in either the dendrites (MAP2, red; B) or axons (TAU protein, red; C). The nucleus is indicated by DAPI (blue) staining in the merged images. D. DIV 18 ESNs were evaluated for the compartmentalization of GRIA1 (green) in either the dendrites (MAP2, white) or axons (TAU protein, red) A–D. Markers of significance are per methods section. Results are averaged among three differentiations.

Evidence of an acute electrophysiological response to glutamate addition suggested the functional expression of ionotropic glutamate receptors (iGluRs). RNA sequencing data from a longitudinal expression profiling experiment was screened for transcripts of iGluRs at DIV −8 (ESCs), DIV 0 (neural progenitor cells) and DIV 16 (developmental stage IV/V neurons; Table 1) [16]. The majority of subunits were either not expressed, or expressed at low levels in ESCs. In neural progenitor cells, transcripts for all AMPAR subunits were present at moderate levels while *Grik5* was strongly upregulated. At DIV 16, ESNs expressed most of the subunits of NMDAR, AMPAR and KAR, with particularly high abundances of *Gria1–4*, *Grik5* and *Grin1*. Single nucleotide polymorphism analysis confirmed that 98.8% of *Gria2* transcripts exhibited the Q/R RNA edit that blocks AMPAR Ca^2+^ permeability (no reads were available at that position at DIV −8 or 0) [18]. The *Adarb1* gene responsible for this edit was also strongly expressed at DIV 16. Immunoblot was used to confirm expression of GRIN1, GRIN2a/b, GRIA1 and GRIK1 protein (Figure 2A) and immunofluorescence at DIV 18 revealed a punctate somatodendritic distribution for GRIN2a/b and GRIA1, consistent with localization to post-synaptic compartments (Figure 2B–D).

**Table 1:** RNA-seq analysis of iGluR expression as a function of neuronal differentiation from mouse embryonic stem cells to glutametegic neurons.

		embryonic stem cells (n = 4)		neural progenitor cells (n = 3)		DIV 16 neurons (n = 5)	
iGluR class	Gene	Average	SD	Average	SD	Average	SD
**AMPAR subunits**	*Gria1*	1.6	1.0	132.4	42.5	3964.2	187.2
	*Gria2*	2.7	1.4	328.5	80.8	7393.6	390.0
	*Gria3*	31.1	5.8	409.8	27.2	2201.8	116.4
	*Gria4*	88.7	23.2	291.3	34.7	6621.4	628.8
**KAR subunits**	*Grik1*	0.3	0.5	150.5	8.3	771.1	42.6
	*Grik2*	4.1	1.9	160.8	27.0	1084.0	64.2
	*Grik3*	301.2	65.5	128.8	13.5	807.7	36.1
	*Grik4*	16.1	6.9	104.8	0.8	641.2	36.2
	*Grik5*	334.0	103.2	1525.6	171.3	4617.2	152.4
**NMDAR subunits**	*Grin1*	313.8	90.6	70.9	14.7	12398.0	514.1
	*Grin2a*	6.0	1.4	3.7	1.3	795.9	67.2
	*Grin2b*	23.0	9.4	11.9	3.6	1577.5	159.2
	*Grin2c*	6.0	4.0	6.0	5.5	14.4	4.4
	*Grin2d*	61.4	11.8	25.2	3.1	359.7	26.2
	*Grin3b*	3.8	0.6	10.7	4.8	9.4	2.9
	*SNAP25*	39.8	7.7	540.0	155.2	24639.3	1592.7
	*Adar1b*	558.2	53.3	376.5	71.0	3070.7	119.8

The average pseudocount and standard deviation (SD) are presented for each iGluR subunit. Single copy is estimated to correspond to 100 pseudocounts in these data.

### Functional verification of iGluR expression

**Figure 3:**
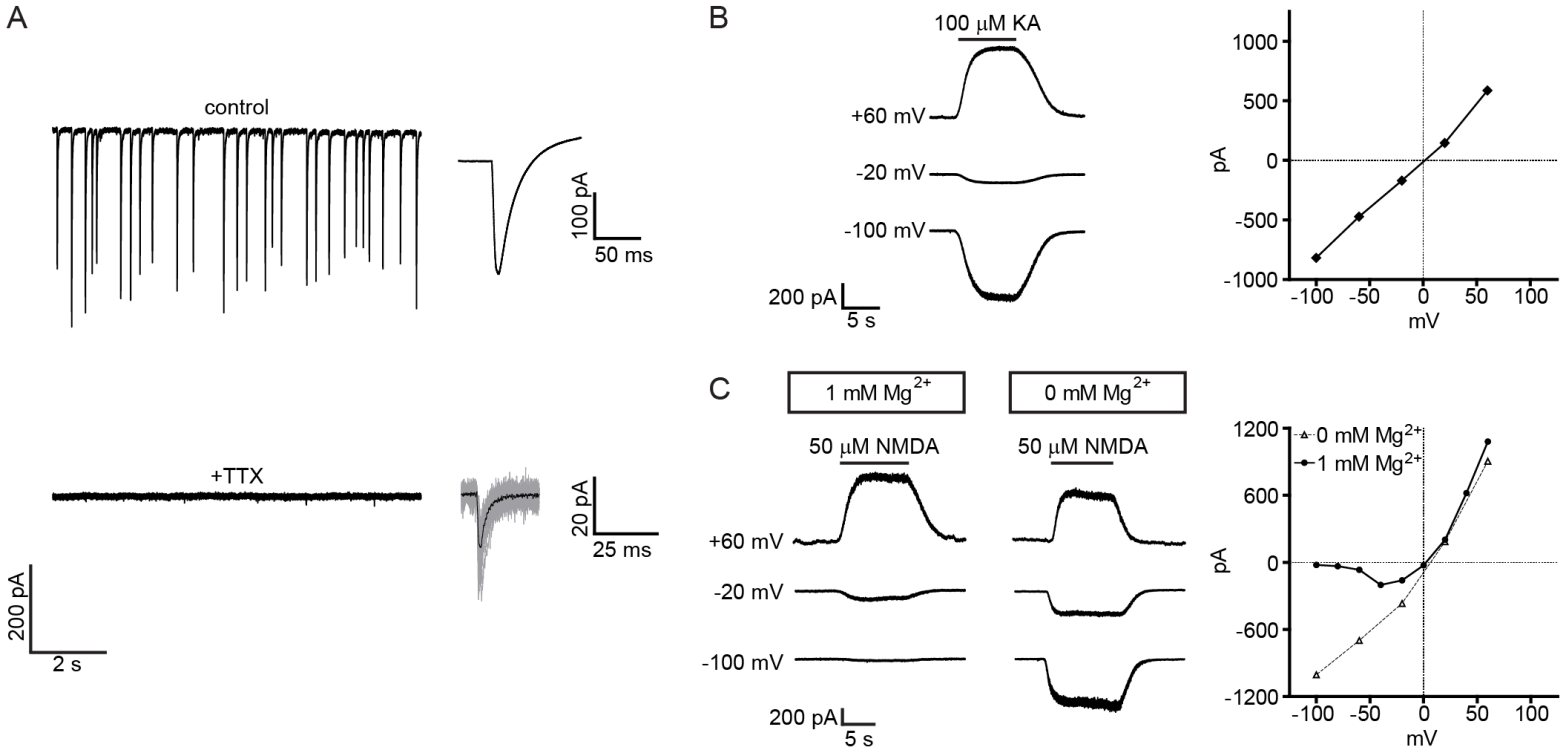
Characterization of functional glutamatergic synaptic activity. A. Representative voltage-clamp recordings from DIV 21 ESNs at resting membrane potential. Robust sEPSCs (top) were eliminated by treatment with TTX (middle), although frequent mESPCs could still be observed. Average sEPSC trace (n = 164 events) and mEPSC trace (n = 52 events) are presented beside recordings. B. Sample traces from a single neuron showing AMPAR-induced currents at three different holding potentials and I-V plot of evoked AMPAR EPSCs (n = 8). Kainate (KA) was used as a low-desensitizing AMPAR agonist. C. Sample traces and I-V plot of evoked NMDAR EPSCs. NMDA-induced currents elicit a voltage-dependent block in the presence of 1 mM Mg^2+^ (right; filled circles; n = 12) that becomes linear in Mg^2+^-free medium (open triangles; n = 8).

All neurons examined at DIV 18+ exhibited spontaneous excitatory post-synaptic currents (sEPSCs) at RMP (n = 28; Figure 3A). To further investigate the functional characteristics of glutamate receptor-mediated currents, the kinetics of miniature EPSCs (mEPSCs) were characterized at DIV 18–24 in the presence of TTX (5 μM) and Mg^2+^ (1 mM) at a holding potential of −80 mV (≥200 events per neuron, n = 12 neurons). Averaged mEPSCs had an amplitude of −18.7±1.05 pA, 10–90% rise time of 1.22±0.20 ms and width at half-amplitude of 1.73±0.09 ms. A double-exponential curve was the best fit in most circumstances, with an average fast component of 1.39±0.17 ms and slow component of 4.28±0.68 ms. The presence of a two-phase decay suggested functional expression of NMDARs and AMPARs. Current-voltage (I–V) plots were generated in whole-cell configuration to further evaluate the voltage-mediated behaviors of AMPARs and NMDARs. While AMPAR currents were linearly proportional to voltage (Figure 3B), the NMDAR I-V curve exhibited a J-shape, consistent with a voltage-block at negative potentials (Figure 3C). Repeating the NMDAR I-V analysis in Mg^2+^-free medium eliminated the voltage sensitivity, confirming that NMDARs exhibit the functional responses necessary for coincidence detector behavior [19].

**Figure 4:**
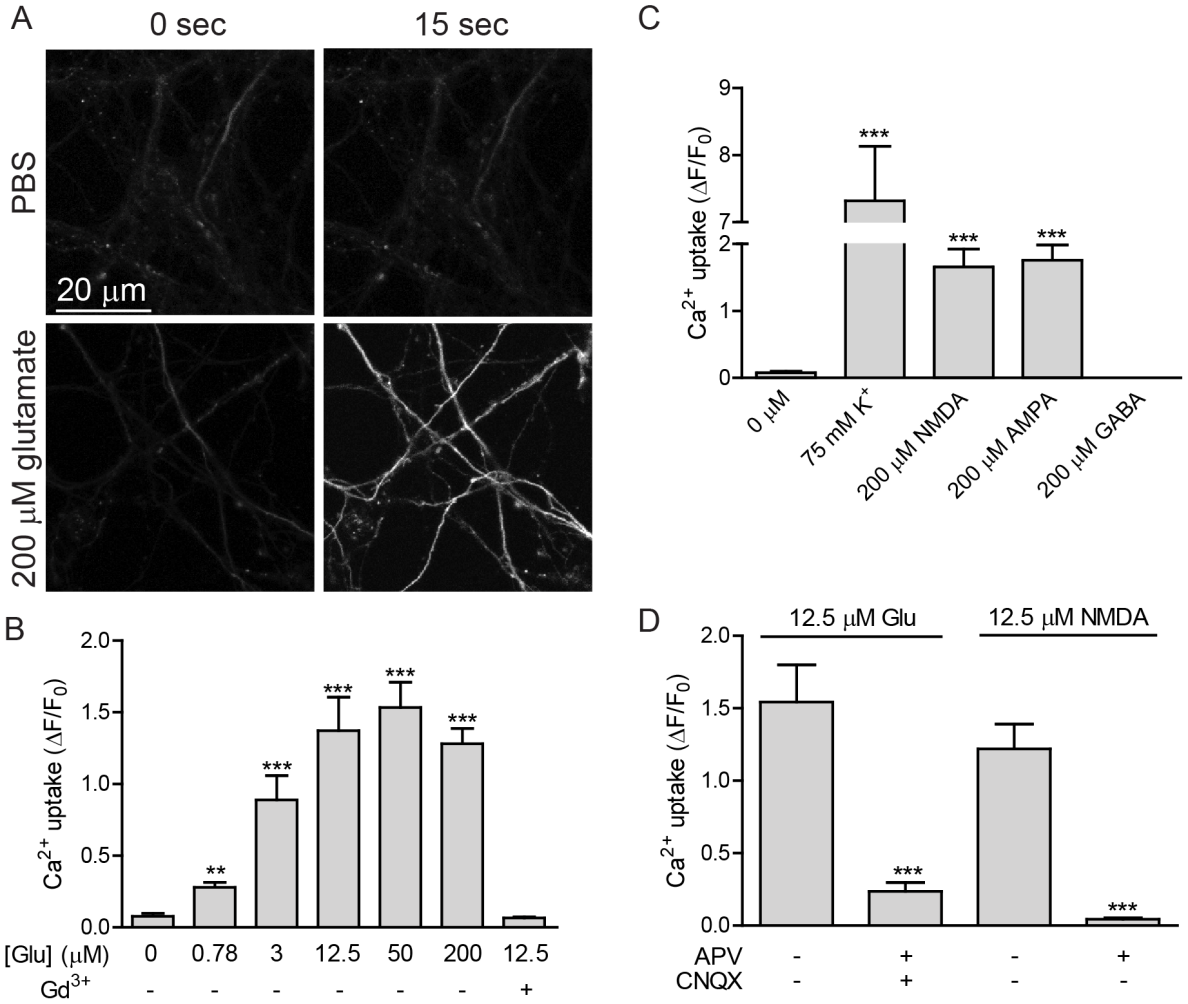
Agonists of GluRs evoke Ca^2+^ uptake. A. ESNs treated with 200 µM glutamate underwent a substantial increase in Ca^2+^-mediated Fluo4 fluorescence within 15 s. B. Glutamate dose-dependent Ca^2+^ uptake. Pretreatment with Gd^3+^ (50 µM) blocked axonal Ca^2+^ uptake. C. Effect of GluR agonists and neuromodulatory chemicals on Ca^2+^ uptake at 30 s after agonist addition. D. Quantification of GluR agonists on Ca^2+^ uptake in the presence (+) and absence (−) of APV (A, 50 µM) and CNQX (C, 10 µM) at 30 s after agonist addition. B-D. Data are expressed as the fluorescence change relative to non-stimulated conditions at 30 s after agonist addition. Markers of significance are per methods section. Results are averaged among three differentiations.

Live imaging of Ca^2+^ uptake was used to further evaluate the functional expression of iGluRs. Glutamate addition evoked a strong Ca^2+^ influx within 15 s (Figure 4A), with a positive correlation between dose and the magnitude of fluorescence (Figure 4B). The Ca^2+^ signal was most robust in axons, suggesting that glutamate treatment was activating voltage-gated Ca^2+^ channels (VGCCs). This was confirmed by pre-treatment with the VGCC antagonist gadolinium (Gd^3+^), which eliminated over 95% of the acute Ca^2+^ signal (Figure 4B). Acute axonal Ca^2+^ uptake was elicited by treatment with the specific iGluR agonists AMPA or NMDA, but not the inhibitory neurotransmitter GABA (Figure 4C), and glutamate-induced Ca^2+^ uptake was efficiently blocked by pre-incubation with APV and CNQX (Figure 4D).

### Acute cytotoxicity is mediated by NMDAR activation in the presence of Ca^2+^

**Figure 5:**
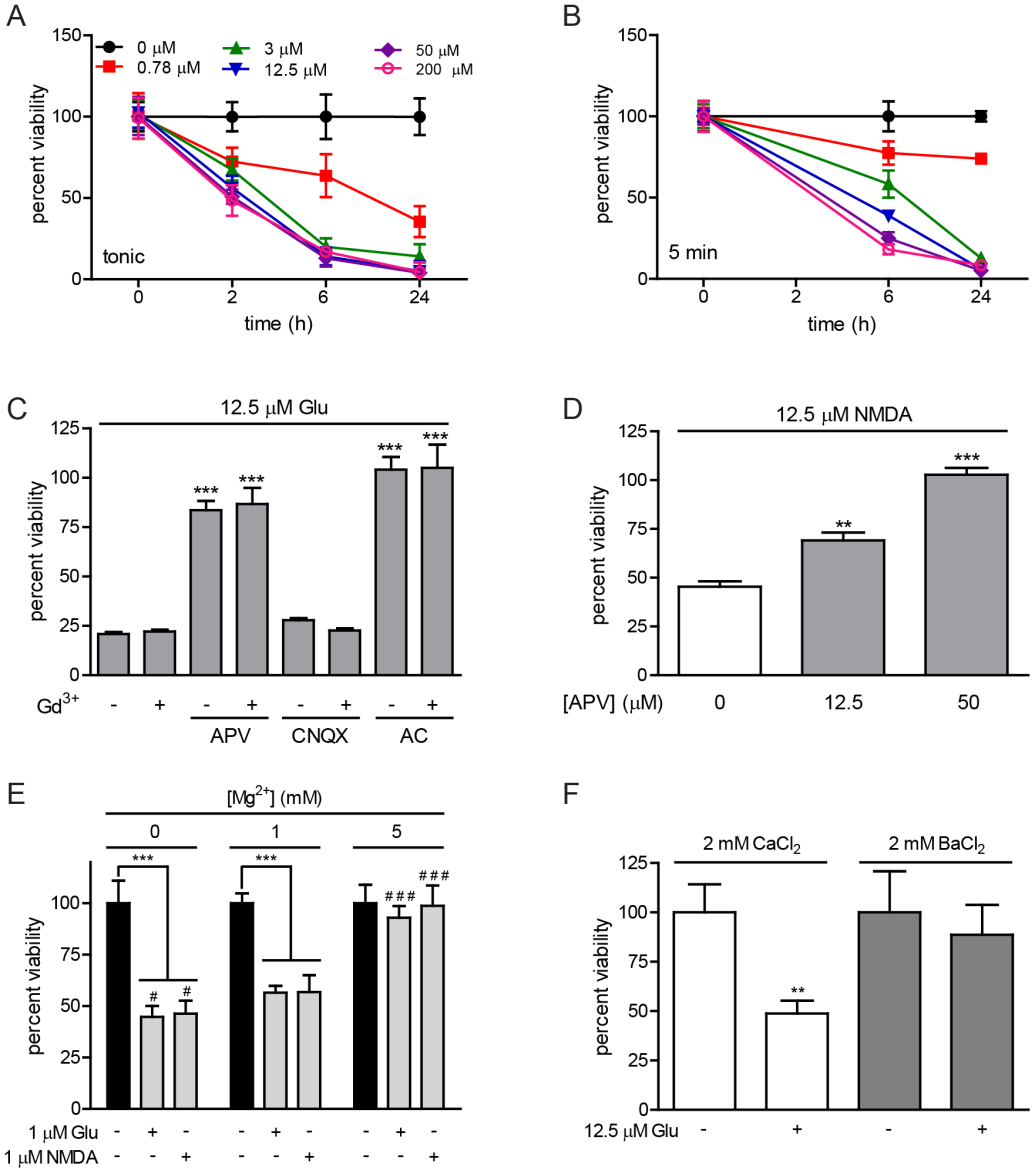
Glutamate treatment causes time- and dose-dependent decreases in neuron viability. A, B. Glutamate dose-dependent reduction in ESN viability over time during a tonic (A) or following a 5-min (B) glutamate treatment. All data points were significantly different from 0 h (P<0.001). C. Quantification of glutamate-induced reduction in neuronal viability in the presence of APV (50 µM), CNQX (10 µM) and/or Gd^3+^ (50 µM). Markers of significance are per methods section relative to glutamate alone. D. APV provides a dose-dependent blockade of cell death at 6 h after a 5 min NMDA treatment. A–D. Data are expressed as percent viability relative to 0 µM glutamate (A−C) or NDMA (D) and are averaged among 8 independent differentiations. (E) Excitotoxicity evoked by a 5 min exposure to glutamate or NMDA is potentiated in 0 mM Mg^2+^ (p<0.05) and inhibited in 5 mM Mg^2+^ (p<0.001) compared to 1 mM Mg^2+^. (F) Glutamate-induced cell death is blocked by substitution of Ba^2+^ for Ca^2+^ (p<0.01). E–F. Data are the average of ≥12 biological replicates.

To determine whether glutamate treatment was neurotoxic to ESNs, metabolic activity was measured at 2, 6 and 24 h after the tonic application of 0.78–200 μM glutamate (Figure 5A). At all doses, metabolic failure was apparent by 2 h and complete by 24 h, with a calculated EC_50_ of 0.41 µM (R^2^ = 0.99). To determine whether transient exposure would also cause toxicity, ESNs were exposed to 0.78–200 μM glutamate for 5 min, followed by incubation in glutamate-free media. As with the tonic treatment, metabolic inhibition was complete at 24 h for all doses above 0.78 μM glutamate, although the rate of cell death was delayed in the pulse treatment (Figure 5B). Tonic addition of NMDA likewise resulted in a dose-dependent loss of ESN viability by 24 h, with a calculated EC_50_ of 0.39 µM (R^2^ = 0.95; Figure S1).

Exposure of ESNs to subtype-specific agonists and antagonists was used to determine whether specific iGluRs were responsible for glutamate neurotoxicity. Whereas CNQX addition was not protective, APV treatment prevented glutamate-induced cell death in a dose-dependent manner (Figure 5C). Notably, inhibition of VGCCs by addition of Gd^3+^ had no effect on viability (Figure 5C). To verify the role of NMDARs in toxicity, we showed that NMDA-induced neurotoxicity was blocked in a dose-dependent manner by APV (Figure 5D). Collectively, these data showed that glutamate-induced neurotoxicity was inhibited by iGluR antagonists in a dose-dependent manner, and further suggested that NMDAR activation was sufficient to cause neuron death.

To further evaluate the role of Ca^2+^ uptake by NMDARs in glutamate-induced neurotoxicity, cell viability assays were conducted by a 5 min treatment with 1 µM glutamate or NMDA in 0, 1 or 5 mM Mg^2+^, followed by wash-out and incubation in fresh NBA-B27 medium for 6 h. Cell death was potentiated in Mg^2+^-free medium, and significantly reduced in 5 mM Mg^2+^ (Figure 5E). A causative role for Ca^2+^ in excitotoxicity was tested by substitution of Ba^2+^ for extracellular Ca^2+^, which conferred full protection against a 5 min treatment with 12.5 µM glutamate (Figure 5F). These data demonstrate that induction of excitotoxicity is dependent on Ca^2+^ uptake, and suggest that methods to reduce the open state of the NMDAR channel concomitantly reduce Ca^2+^-mediated excitotoxicity.

### Glutamate treatment evokes morphologic and genetic markers of neurotoxicity

**Figure 6:**
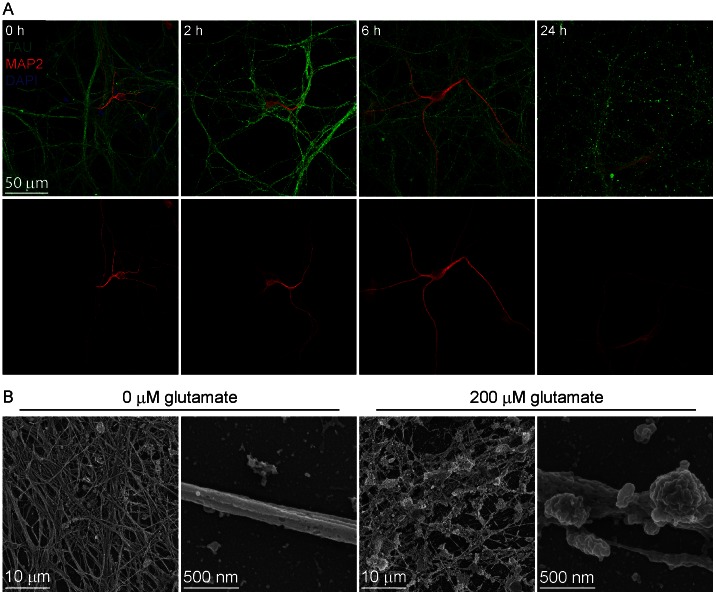
Effects of glutamate treatment on neuron morphology. A. Time-course evaluation of varicosity formation and neuronal degeneration within ESNs treated with 12.5 µM glutamate. At indicated time points axons and dendrites were visualized with TAU (green) and MAP2 (red), respectively. The top panel is a merge of axon, dendrites, and nuclear labeling (blue) with the bottom panel displaying dendrites alone for clarity. B. Representative SEM micrographs of neurons 6 h after addition of vehicle (left panels) or 200 μM glutamate (right panels).

Glutamate-induced changes in neurite morphologies were characterized by immunofluorescent staining for the dendritic/somatic marker MAP2 and the axonal marker TAU protein. Although there was no apparent morphological evidence of neurotoxicity at 2 h, varicosity formation in axons increased 370% at 6 h (p<0.05) and 1,100% at 24 h (p<0.01), and axonal degeneration was visually extensive by 24 h (Figure 6A, upper panels). No gross changes in dendrite morphology were apparent, although MAP2-staining was less intense at 6 and 24 h (Figure 6A, lower panels). Neurite degeneration was further evaluated using scanning electron microscopy (SEM) of neurons treated with 200 μM glutamate for 6 h (Figure 6B). In contrast to the well-defined axodendritic processes in control neurons, glutamate evoked significant changes in morphology, including neurite fragmentation and loss, somatic blebbing, varicosity formation, ruffled membranes and focal neurite swelling.

**Figure 7:**
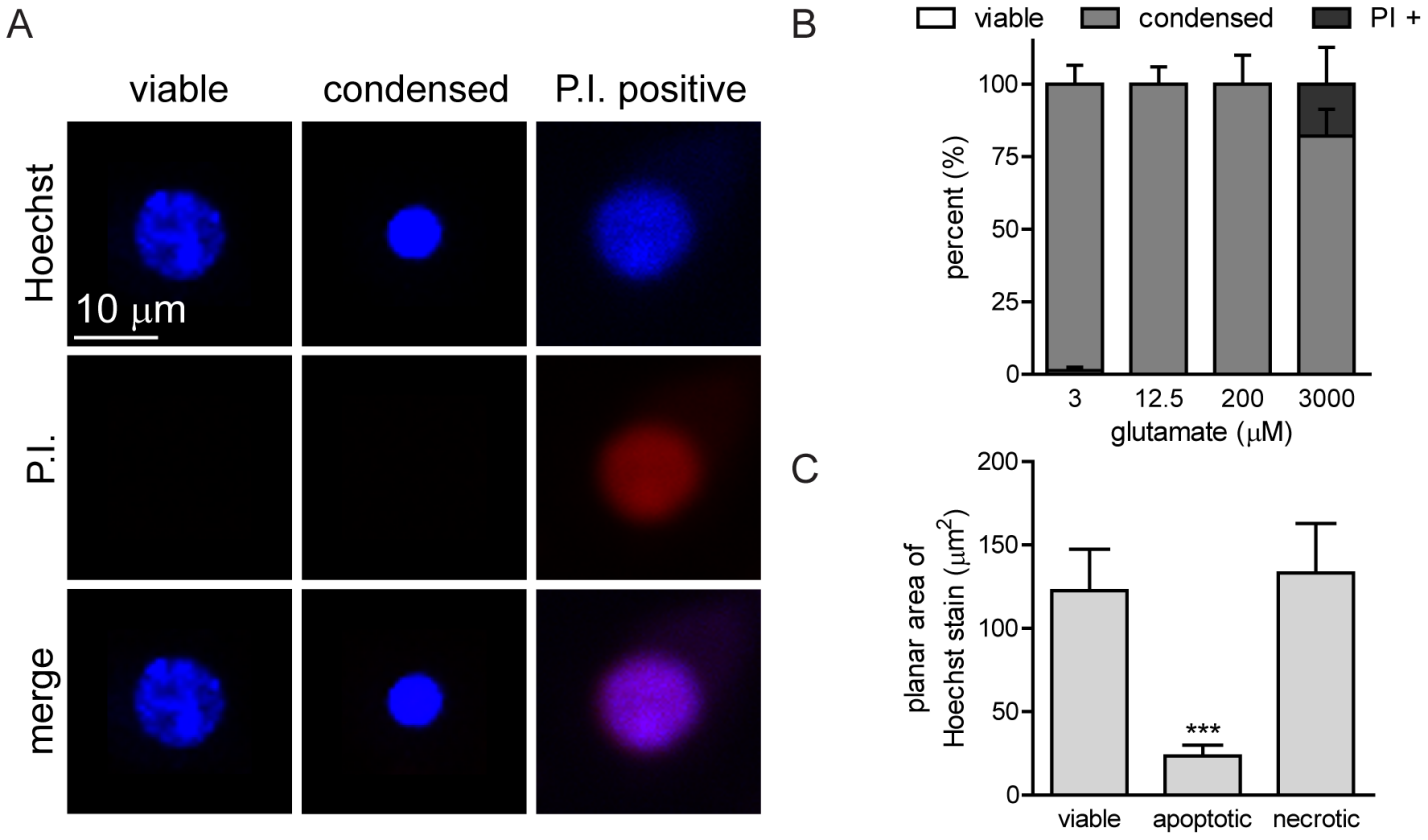
Glutamate activation of cell death pathways. A. Representative photomicrographs of viable, apoptotic or necrotic neurons stained with Hoechst 33342 (blue) and propidium iodide (PI, red). Results are averaged from three independent experiments. B. Quantification of dose-dependent cell death over time (n≥200 nuclei per dose). C. The planar area of Hoechst 33342 stain was calculated from the average nuclear diameter of apoptotic (3.5 µM glutamate) or necrotic neurons (3000 µM glutamate). Markers of significance are per methods section. B, C. Results are from >600 neurons per differentiation, averaged over three 3 independent differentiations.

The pairing of fluorescent membrane-permeant and impermeant nuclear dyes was used to evaluate the mode of excitogenic cell death (Figure 7A) [20]. Tonic addition of 3–200 μM glutamate resulted in pyknotic, PI-negative nuclei at 2 h, indicative of apoptosis (Figure 7B). Evidence of primary necrosis (non-pyknotic, PI-positive nuclei) was first observed with 3000 μM glutamate, which produced a mixture of necrotic and apoptotic neurons (Figure 7B,C).

QPCR was used to confirm that transcripts associated with cell stress and death were upregulated at 6 h after tonic addition of 200 μM glutamate (Table 2), including caspases and genes associated with the regulation of apoptotic and autophagic cell death.

**Table 2:** QPCR analysis of neuronal and stress response genes.

Gene	normalized log_2_ (FC)	function
*Grin1*	0.39	synaptic activity
*Snap25*	−0.52	
*Casp4*	2.96	cell stress/death
*Casp6*	2.15	
*Casp12*	3.03	
*Cdk1*	3.11	
*CflaR*	2.54	
*Dap1*	2.84	
*Dapk2*	4.18	
*Dram1*	2.63	
*Fadd1*	1.98	
*Pawr*	3.49	
*Traf1*	5.02	

Log_2_ fold-change (FC) of select transcripts after 6 h of glutamate exposure compared to untreated neurons. Control or glutamate-treated samples were normalized to the neuron-specific marker *Tubb3* (β3-tubulin).

### Inhibition of glutamate-induced neurotoxicity

Protection against glutamate-induced neurotoxicity was evaluated in ESNs plated in 24–48 well dishes. Gd^3+^ was not used in these experiments because it did not appear to have an effect on neurotoxicity and to avoid masking an intrinsic neuroprotective effect induced by synaptic activity and/or possibly modulated by treatments.

**Figure 8:**
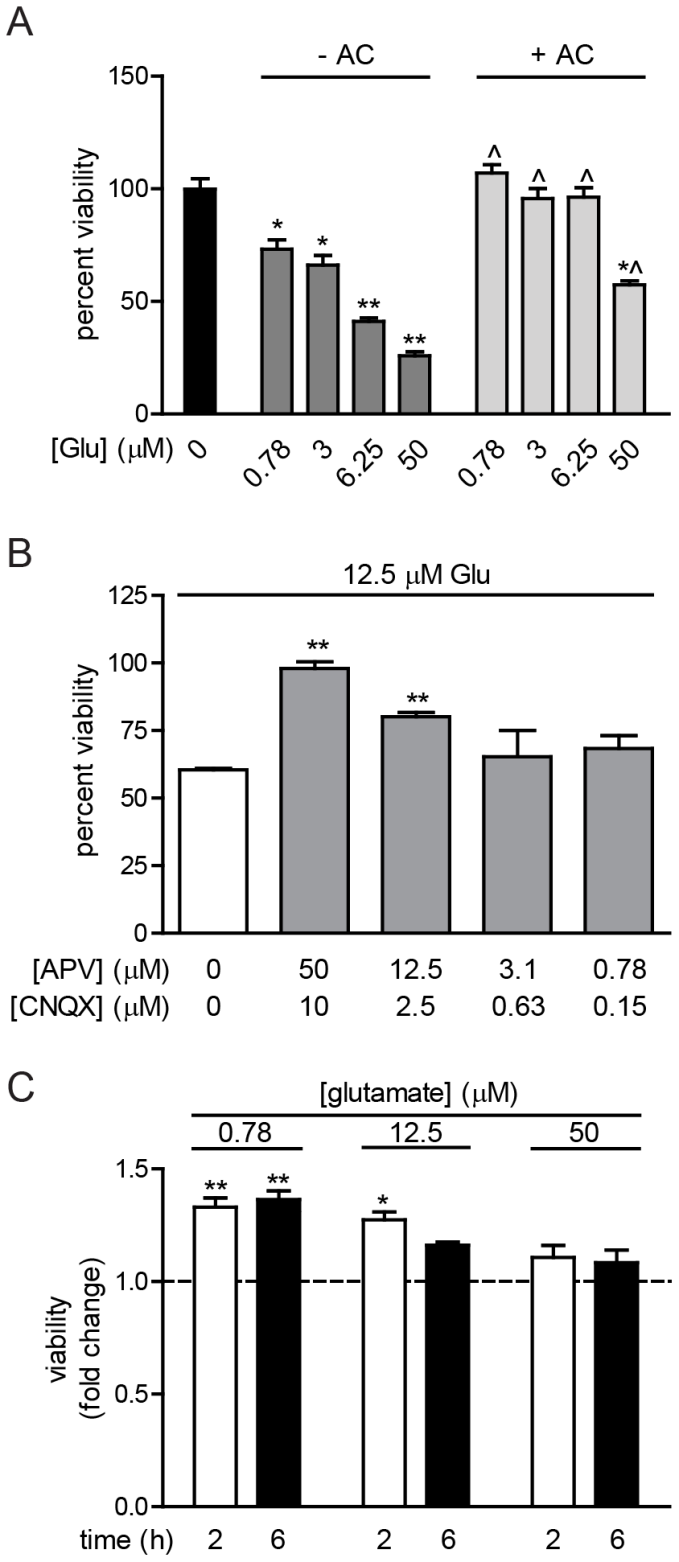
Evaluation of small molecule inhibitors for glutamate-induced neurotoxicity. A. Addition of APV (A, 50 µM) and CNQX (C, 10 µM) blocks glutamate-induced toxicity at 6 h. ? indicates P<0.01 between glutamate only and glutamate supplemented with APV/CNQX. (+) and (−) indicate the presence or absence of glutamate or APV/CNQX. B. Dose-dependent blockade of glutamate-induced cell death by APV and CNQX at 6 h. A,B. The data are expressed as percent viability relative to 0 µM glutamate. C. Prophylactic and co-administration of an NTF cocktail reduced neuron death at 2 and 6 h after glutamate addition. Data are expressed as the fold change in viability of the NGF-pretreated ESNs relative to the glutamate-treated ESNs at the indicated concentration. The dashed line represents the normalized viability of glutamate-treated ESNs at the indicated concentrations. A–D. The data are combined from 6 independent differentiations.

ESN viability was measured 6 h after administration of 50 μM APV +10 μM CNQX (1xA/C) and 0.78–50 μM glutamate. 1xA/C conferred full protection against excitotoxicity up to 12.5 μM glutamate and resulted in reduced toxicity at 50 μM glutamate (Figure 8A). In the reverse experiment, neurons were administered 12.5 μM glutamate with dilutions of 1xA/C, and neuron viability was measured at 2 h (Figure 8B). Whereas 1xA/C conferred full protection against 12.5 μM glutamate, a four-fold decrease in A/C concentration reduced viability by 20%, and subsequent dilutions had no significant protective effect.

Next, we evaluated the ability of a cocktail of neurotrophic factors (NTFs) to delay glutamate-mediated neurotoxicity. Although the prophylactic efficacy of these NTFs has not been described with respect to excitogenic injury, activation of cognate neurotrophic receptors has been reported to be neuroprotective in primary neuron cultures [21]. NTFs were chosen based on three criteria: their efficacy had not previously been evaluated in excitotoxic injury; several of the cognate receptors have been implicated in excitotoxic neuroprotection; and ESNs express transcripts for the cognate receptors [11], [21]. ESNs were pre-treated with NT3, BNDF, GNDF and CNTF for 16 h, and neuron viability was measured at 2, 6 and 24 h after addition of 0.78, 12.5 and 50 μM glutamate. NTF prophylaxis conferred partial protection against tonic glutamate treatment in a dose-dependent manner at 2 and 6 h (Figure 8C). No significant protective effect was apparent at 24 h (not shown), suggesting a limited efficacy during tonic dosing.

## Discussion

### Overview

An ideal *in vitro*-derived, cell-based platform for excitotoxicity research would combine the verisimilitude of primary neurons with the scalability and flexibility of continuous cell lines. The objectives of this study were to determine whether ESNs were sensitive to glutamate-induced excitotoxicity, with morphologic and cellular outcomes similar to those reported in primary neurons; to characterize the relationship between glutamate dose and neuron fate; and finally to evaluate the suitability of ESNs for moderate-throughput screening approaches by evaluating the neuroprotective efficacy of several small molecules.

### Characterization of functional iGluR expression

Transcriptome profiling indicated that ESNs express nearly all iGluR subunits at DIV 14. *Grin1* was the most abundant subunit, expressed at 70.2% the level of *SNAP25* (the most abundant pre-synaptic protein, and the 5^th^ most abundant transcript overall [11]), and representing 0.045% of the total transcriptome. Evaluation of protein expression identified subunits for each iGluR class, with dendritic and somatic expression of GRIN2A/B and GRIA1. Since *Gria2* is the most abundant AMPAR subunit, and 98% of *Gria2* transcripts exhibit the Q to R edit, it is probable that that the vast majority of AMPARs are impermeable to calcium. Single-channel recording is currently underway to confirm this and to determine whether the subunit composition of AMPARs change in response to excitotoxic stimuli [22].

Electrophysiological characterization revealed that DIV 18–24 ESNs exhibit neuron-like electrical behaviors, with a characteristic resting membrane potential, intrinsic electrical activity and the ability to express repeated action potentials in response to a depolarizing current. Spontaneous EPSCs and mEPSCs were regularly measured by DIV 18+, indicative of trans-synaptic communication, and I-V curves confirmed. Electrophysiology and live imaging studies were used to confirm the functional expression of AMPARs and NMDARs, and bath application of glutamate to whole-cell patched ESNs resulted in bursts of action potentials followed by a sustained membrane depolarization. A similar phenomenon has been attributed to a persistent Ca^2+^ current in cultured hippocampal neurons exposed to glutamate [23]–[25]. It will be particularly interesting to correlate electrophysiological measurements of the neuronal response to glutamate with viability assays, since we found that glutamate toxicity is induced within 5 min.

### Glutamate-induced toxicity is mediated by Ca^2+^ uptake through activated NMDARs

In viability assays, bath application of glutamate or NMDA was neurotoxic, with sub-micromolar EC_50_ values measured within 6 h. Surprisingly, a 5 min exposure to glutamate was as toxic as a tonic exposure at 24 h, demonstrating that the mechanisms responsible for neuronal death are initiated rapidly and, once initiated, are not rescued by glutamate washout. This is consistent with findings in dissociated hippocampal neurons, in which irreversible initiation of cell death occurred within 15 min after addition of 10 µM glutamate [26]. Although inhibition of AMPAR/KAR activation did not alter glutamate toxicity, inhibition of NDMAR activation conferred resistance to glutamate, implicating NMDAR in excitototoxicity. Using a combination of electrophysiology and cell viability assays, we showed that modulating Ca^2^ uptake by changing the open period of NMDAR alters the progression of excitotoxicity in ESNs. These data confirm the role of NMDARs in glutamate-induced toxicity and are consistent with a pathogenic role for Ca^2+^. The ability of ESNs to replicate the complex gating of NMDARs suggests that they may be well-suited for exploration of the cellular and molecular components of NMDA-mediated excitotoxicity. Building on these findings, experiments are underway to determine whether the NMDAR localization alters glutamate-induced toxicity and to correlate transcriptomic, electrophysiologic and kinomic changes to the progression of neurotoxicity.

It is estimated that the extracellular glutamate concentration in the CNS is 0.6 µM, with as little as 2 µM glutamate sufficient to injury certain brain regions [27]. In *in vitro* assays, ESNs appear more sensitive than the most commonly used primary neuron cultures and neurogenic cell lines (Table 3). One possible reason for this difference may be that primary cultures contain varying proportions of excitatory neurons, inhibitory neurons and glia. Such a complex population is likely to introduce variability in measurements of glutamate sensitivities and excitogenic responses. In contrast, ESNs are predominantly glutamatergic, offering a narrow range of biological responses. Though ESNs may not exhibit emergent behaviors arising from the interaction of multiple neural cell types [28], in exchange they offer the facile application of genetic, biochemical and cell biology tools in a highly enriched and physiologically relevant culture.

**Table 3:** Comparison of EC_50_ values for excitotoxicity cell models.

Cell Type	Species	EC_50_(μM)	Assay	Reference
ESN	mouse	0.41	metabolic activity	this manuscript
Cerebellar granule	mouse	10	nuclear staining	[36]
Cerebellar granule	rat	500	metabolic activity	[37]
Hippocampal	mouse	5	nuclear staining	[38]
Cortical	mouse	20	metabolic activity	[39]
PC12	rat	>1000	metabolic activity	[40]
SH-SY5Y	human	>1000	metabolic activity	[41]
NT2-N	human	10	metabolic activity	[42]

EC_50_ values were obtained or calculated from primary data at 24 hours post-exposure.

### Excitogenic modes of cell death involve the strongly dose-dependent presentation of apoptotic and necrotic markers

Within 6 h of glutamate treatment, axons became visibly varicosed and fragmented. Axonal degeneration has been associated with mitochondrial disruption and activation of calpains and caspases in other injury modalities, suggesting that excitogenic axon degeneration is the result of traditional apoptotic mechanisms [29]. The focal neurite swelling, membrane ruffling and somatic blebbing observed at 6 h after glutamate treatment in SEM micrographs are also consistent with apoptotic progression and dysregulation of ion pumps [30]. To determine whether these morphologic markers were evidence of apoptosis, we used nuclear condensation and the acute loss of membrane integrity as markers of apoptosis and necrosis, respectively [31]. ESNs exclusively exhibited apoptotic markers at glutamate concentrations up to 200 μM, while 3000 μM resulted in a mixture of necrosis and apoptosis. The appearance of multiple modes of cell death is consistent with *in vivo* and *in vitro* reports of excitotoxic outcomes [8], [9], [32]–[34], and raises the possibility that excitogenic progression involves the dose-dependent induction of multiple cell death pathways, with the degree of the stimulus determining which mode is preeminent.

### Potential for moderate-throughput therapeutic screening and mechanistic discovery

The scalable yield of ESNs mitigates problems with cost and reproducibility that have hindered moderate-throughput drug screening approaches based on primary neurons and neurogenic cells. The ability to plate ESNs in a variety of formats, including multi-well plates, also allows for moderate-throughput screening of therapeutics [11]. This was first demonstrated in section 3.2, in which mitochondrial function was evaluated by plate reader using ESNs plated in 24-well dishes. As an additional proof-of-concept, we demonstrated the potential for therapeutic screening by testing the ability of two small-molecule cocktails to rescue or attenuate glutamate-induced neurotoxicity. NTF prophylaxis conferred dose-dependent protection against tonic glutamate treatment, suggesting that optimization of glutamate treatment conditions may allow the sensitive detection of prophylactic and therapeutic neuroprotective candidates. The scalability and versatility of ESNs provides a strong foundation for the development of cell based screening platforms that will not only provide homogeneity among samples and experiments, but also significantly reduce the ethical and logistical burden of using primary neurons and animal models.

### Summary

Neuronal responses to excitotoxicity are highly dependent on the intensity and duration of the excitatory insult, the complement of glutamate receptors expressed, and the localization of the functional receptors [35]. Primary neuron cultures typically suffer from origin-specific variations in the relative proportions of excitatory neuron subtypes, inhibitory neurons and glial cells; this variability is likely to introduce variability in measurements of glutamate sensitivities and excitogenic responses. In principle, the highly enriched glutamatergic composition of ESNs offers several advantages over complex cell populations: a narrower range and variability of biological responses; minimal contributions by inhibitory neurons that might reduce NMDAR activation; and few glial cells to absorb exogenous glutamate and release neurotrophic factors. ESNs were shown to replicate known responses of primary neurons to glutamate, including toxicity mediated by Ca^2+^ influx through NMDARs and physiological behaviors of post-synaptic iGluRs. The high degree of purity and scalable derivation facilitates the use of ‘omics’-based approaches to evaluate short- and long-term cellular responses to excitotoxicity. These data suggest that ESNs are a novel cell model for excitotoxicity research that combines the verisimilitude of primary neurons with the flexibility of continuous cell lines. The identification of an ESN-based excitotoxicity model should enable detailed biochemical and molecular processes involved downstream of NMDAR-mediated Ca^2+^ influx that are difficult or impossible to address in neurogenic cells or primary neurons. This model is currently being used to conduct mechanistic investigations into excitotoxic injury, with the objective of identifying assays and points of intervention to facilitate therapeutic screening.

## Supporting Information

Figure S1 **NMDA treatment causes a dose-dependent reduction in ESN viability at 24 h.** The data are expressed as fold-change relative to 0 µM NMDA. Markers of significance are per methods section. The data are combined from 4 independent experiments.
(TIF)

Table S1 **QPCR primer list.** Gene names, primer orientations, primer sequences, and length of amplicons for QPCR reaction.
(DOCX)
