# Supplementary figures and images for: Novel Application of Stem Cell-Derived Neurons to Evaluate the Time- and Dose-Dependent Progression of Excitotoxic Injury
Source: PLoS One. 2013 May 14;8(5):e64423. doi: 10.1371/journal.pone.0064423 (PMC3653859; doi:10.1371/journal.pone.0064423)

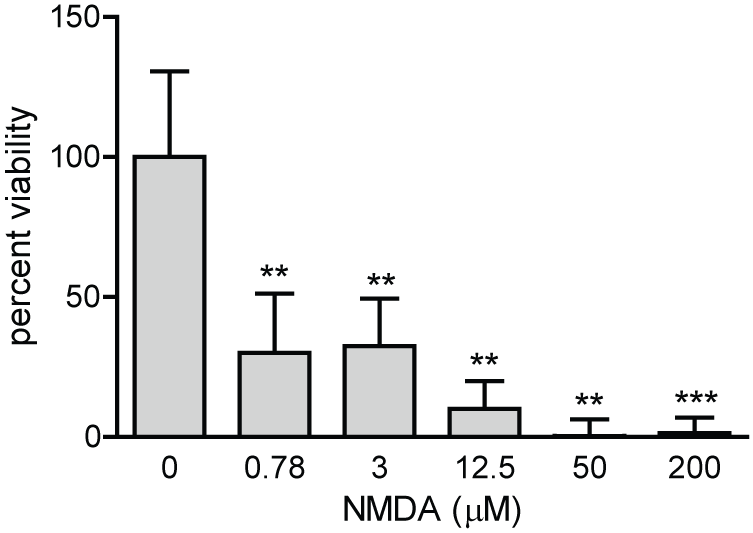

Supplement: Figure S1 — NMDA treatment causes a dose-dependent reduction in ESN viability at 24 h. The data are expressed as fold-change relative to 0 µM NMDA. Markers of significance are per methods section. The data are combined from 4 independent experiments. (TIF) [file pone.0064423.s001.tif]
