# Supplementary material for: Novel Application of Stem Cell-Derived Neurons to Evaluate the Time- and Dose-Dependent Progression of Excitotoxic Injury
Source: PLoS One. 2013 May 14;8(5):e64423. doi: 10.1371/journal.pone.0064423 (PMC3653859; doi:10.1371/journal.pone.0064423)
Supplement: Table S1 — QPCR primer list. Gene names, primer orientations, primer sequences, and length of amplicons for QPCR reaction. (DOCX) [file pone.0064423.s002.docx]

| **gene** | **primer orientation** | **Sequence (5' − 3')** | **amplicon size (bp)** |
| --- | --- | --- | --- |
| *Casp4* | forward | AGCGTTGGGTTTTTGTAGATGC | 179 |
|  | reverse | CCTTGTGAACTCTTCAGGGGA |  |
| *Casp6* | forward | AAAAGTAGGGAAGTGTTCGATCC | 152 |
|  | reverse | CGAGTCAGGTTGTCTCTGTCTG |  |
| *Casp12* | forward | ATGCTGGATTGGCCCATGAAT | 172 |
|  | reverse | AGACGTGTTCGTCCCTCCTT |  |
| *Cdk1* | forward | AGGTACTTACGGTGTGGTGTAT | 85 |
|  | reverse | CTCGCTTTCAAGTCTGATCTTCT |  |
| *Cflar* | forward | TGGCTGAATTGCTCTACAGAGT | 105 |
|  | reverse | CCAGGTGAGGGTTTCTGCG |  |
| *Dap1* | forward | GATGCGGATTGTGCAGAAACA | 123 |
|  | reverse | GCAATAACGCCAGAGATGAACA |  |
| *Dapk2* | forward | GCTCACTTTGATCTCAAGCCA | 240 |
|  | reverse | GGACGCTCCACTTAGAAGGATA |  |
| *Dram1* | forward | CATCTCCGCTGTTTCGTGC | 84 |
|  | reverse | GGATTCCATTCCAGCTTGGTTA |  |
| *Fadd1* | forward | TGGCTGAATTGCTCTACAGAGT | 105 |
|  | reverse | CCAGGTGAGGGTTTCTGCG |  |
| *Grin1* | forward | TCCCAACGACCACTTCACTC | 95 |
|  | reverse | AGTAGATGGACATTCGGGTAGTC |  |
| *Pawr* | forward | AAGCGAGAGGATGCCATCAC | 248 |
|  | reverse | TTTTCCAAGGTGCTACTTGAGG |  |
| *SNAP25* | forward | ATCCGCAGGGTAACAAATGATG | 83 |
|  | reverse | CGGAGGTTTCCGATGATGC |  |
| *Traf1* | forward | ATGAGGATCGGATCTGTCCTAAA | 97 |
|  | reverse | GCTACATCAGAGTGAACCTTCTC |  |
| *Tubb3* | forward | CCCAGCGGCAACTATGTAGG | 143 |
|  | reverse | CCAGACCGAACACTGTCCA |  |
